# Reduction in *Aedes aegypti* Population After a Year-Long Application of Targeted Sterile Insect Releases in the West Valley Region of Southern California

**DOI:** 10.3390/insects16010081

**Published:** 2025-01-15

**Authors:** Solomon Kibret Birhanie, Jacob Hans, Jennifer Thieme Castellon, Ale Macias, Rubi Casas, Huy Hoang, Deanna Mormile, Kecia Pitts-Love, Michelle Q. Brown

**Affiliations:** West Valley Mosquito and Vector Control District, 1295 East Locust St, Ontario, CA 91761, USA

**Keywords:** *Aedes aegypti*, population reduction, X-ray irradiated mosquitoes, integrated vector management

## Abstract

*Aedes aegypti* is of great public health concern because of its vectorial capacity to transmit various arboviruses such as Zika, yellow fever, dengue, and chikungunya. In California, its expanding geographic distribution has been unrestrained. This urgently calls for innovative tools such as the use of sterile insect technique (SIT) to strengthen invasive *Aedes* control. This study aimed to determine the efficacy of combining an SIT application into integrated vector management (IVM) strategies in invasive *Aedes* control in the West Valley region of southern California. A total of 25 *Aedes* hotspots, grouped into two cohorts—sites that received SIT treatment only (*n* = 9) and sites that received both SIT and In2Care^®^ Mosquito Stations (*n* = 16)—were selected for this study. Biweekly, X-ray irradiated male *Ae. aegypti* mosquitoes were released between April and November 2024. Data from weekly BG Sentinel-2 traps were utilized to compare the mosquito densities between the pre-intervention (2023) and intervention (2024) periods for both cohorts. In addition, a subset of samples (*n* = 50) of irradiated male mosquitoes were placed in a separate cage with freshly emerged unirradiated female mosquitoes to evaluate the level of sterility of the eggs from these mosquitoes. *Aedes*-related service requests made by the District’s residents were also compared between the pre-intervention and intervention periods to indirectly determine the effect of optimizing the control strategies on overall mosquito problems. A total of 106,608 sterile male *Ae. aegypti* were released between April and November 2024. The cohort with the SIT only application showed a 44% reduction in the number of female *Ae. aegypti* per trap-night during the intervention period compared with the pre-intervention period. At sites with In2Care Mosquito Stations that also received SIT treatment, the mosquito density dropped by 65% during the intervention period compared with the pre-intervention period. The number of *Aedes*-related service requests during the intervention year (*n* = 367) was 45% lower than the pre-intervention year (*n* = 656). Over 99.6% eggs collected from female mosquitoes mated with irradiated males did not hatch, indicating a high level of sterility of the irradiated males utilized for our SIT application. Here, we demonstrated the potential of SIT when combined with IVM strategies such as In2Care^®^ Mosquito Stations, offering a holistic approach to reducing the public health risks associated with *Aedes*-borne diseases.

## Simple Summary

This study highlights the significant impact of using targeted sterile insect technique (SIT) in reducing the population of *Aedes aegypti*, a major vector of dengue, chikungunya, yellow fever, and Zika viruses. Over a year-long SIT application in the West Valley region of southern California, we observed a dramatic decline in *Ae. aegypti* mosquito populations, providing a compelling case for SIT as an innovative and sustainable vector control strategy when utilized along with the existing integrated vector management tools. Our work highlights the use of targeted SIT implementation to address the growing challenge of vector-borne diseases in urban settings, especially in small-to medium-sized vector control districts.

## 1. Introduction

*Aedes aegypti* is of great public health concern because of its vectorial capacity to transmit various arboviruses such as Zika, yellow fever, dengue, and chikungunya [1]. In California, its expanding geographic distribution has worried the public [2]. Between 2016 and 2023, over 1200 travel-associated dengue cases were documented in California [3], indicating the risk of local transmission due to the presence of a competent vector. In 2023, two locally acquired dengue cases were reported for the first time in southern California [4]. Additionally, a total of 15 locally acquired dengue infections were documented in 2024, indicating the increasing risk of local transmission occurring regularly [4]. This indeed urgently calls for innovative tools to strengthen the existing integrated vector management (IVM).

The existing IVM utilizes various strategies such as source reduction, biological control (e.g., larvivorous fish), chemical treatment, and community engagement to mitigate the expanding distribution of invasive mosquitoes. In2Care^®^ Mosquito Stations, which combine pyriproxyfen autodissemination with the entomopathogenic fungus *Beauveria bassiana* to reduce the emergence of *Aedes* mosquitoes and subsequently kill the contaminated adult females [5], have been recently added to complement IVM strategies, especially in urban areas with multiple small, cryptic breeding sites [6]. The most common *Ae. aegypti* breeding habitats in California include backyard containers (e.g., discarded cans, bottles, and buckets), flowerpots, fountains, and bird baths [6]. Eggs of these mosquitoes can survive desiccation for a longer time and hatch when containers fill with water. However, while existing IVM tools continue to moderately suppress invasive mosquito breeding, new innovative tools are required to effectively control the spread of these mosquitoes in diverse settings. Sterile insect technique (SIT) is therefore emerging as a promising and eco-friendly method for controlling *Aedes aegypti* populations [7,8], especially when used along with the existing IVM strategies.

The SIT has been successfully used for decades to control several insect pests such as the Mediterranean fruit fly [9], New World screwworm [10], Mexican fruit fly [11], tsetse fly [12], and pink bollworm [13]. Due to the cryptic breeding habitats of *Aedes* mosquitoes [14], the sterile insect technique (SIT) has gained momentum as a potential biological control strategy to fight these invasive mosquitoes.

The SIT involves mass-rearing male mosquitoes, sterilizing them through irradiation or genetic modification, and then releasing them into the wild. Unlike genetic modification (GM)-based SITs that involve the introduction of engineered genes into male mosquitoes to render them sterile or lethal to their offspring, irradiation-based SIT utilizes ionizing radiation, such as X-rays, to sterilize the male mosquitoes [15]. The radiation damages the reproductive cells of the male mosquitoes, rendering them sterile without affecting their overall fitness for mating [16] The sterile males are then released into the wild, where they compete with wild males for female mates. When wild females mate with sterile males, no viable offspring are produced, gradually reducing the mosquito population.

The success of the SIT depends on several factors including the release of a sufficient number of sterile males, ensuring that they are competitive with wild males for mating opportunities, and sustaining multiple releases over time. Logistics is another critical factor that affects the scalability of SIT-based vector control interventions, especially in area-wide applications. Targeted application, on the other hand, requires limited resources and focuses on specific hotspots where mosquitoes pose the greatest risk. This approach is especially preferable for small- to medium-sized vector control agencies, since it prioritizes areas with high mosquito density or disease risk, unlike area-wide treatment. Since *Ae. aegypti* mosquitoes disperse short distances [17], released sterile mosquitoes will remain in the targeted area, increasing the likelihood of sterile males mating with wild females in the targeted hotspots to result in the desired outcome.

Here, we report on the first-year outcome of targeted sterile insect releases in southern California to mitigate invasive *Ae. aegypti* mosquitoes. The study aimed to evaluate the impact of sterile insect releases on the overall *Ae. aegypti* population reduction in historical *Aedes* hotspots.

## 2. Materials and Methods

### 2.1. Study Area

**Figure 1:**
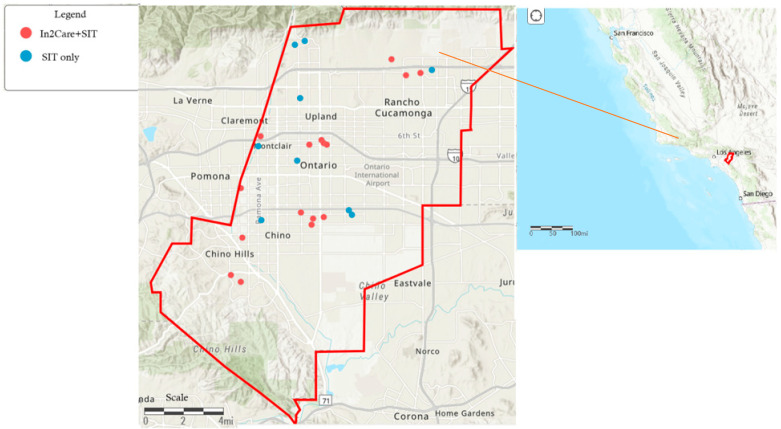
Study area of the West Valley Mosquito and Vector Control District, southern California. Red dots indicate sites that received both SIT treatment and an In2Care^®^ Mosquito Station and; blue dots denote sites that received SIT treatment only.

This work was conducted in the West Valley Mosquito and Vector Control District (WVMVCD) in southwestern San Bernardino County, California (Figure 1). The District covers 544 km^2^ and serves over 600,000 residents in the cities of Chino, Chino Hills, Montclair, Ontario, Rancho Cucamonga, Upland, and surrounding unincorporated county areas. The region exhibits a semi-arid climate, with hot summers and mild winters. The summers (June to September) are characterized by temperatures that can reach up to 43 °C. During the winter (December to February), the temperatures drop to as low as 5 °C, with minimal rainfall (average annual rainfall = 381 mm) (www.usclimatedata.com, accessed on 4 December 2024). The mosquito season lies in the summer. Peak *Ae. aegypti* population occurs between July and September [18].

### 2.2. Preparation for Sterile Insect Release

Locally obtained *Ae. aegypti* mosquitoes were reared in the WVMVCD insectary. The details of the mosquito rearing procedure have been documented in our recent work [19]. In brief, mosquito eggs were hatched in a flask with yeast infused 400 mL water and kept under 600 mmHg negative pressure for one hour. Once hatched, the flask was emptied into a large tub (35.9 cm × 19.4 cm × 12.4 cm) with 2 L of tap water and 0.44 g alfalfa rabbit food pellets. A further 0.44 g of alfalfa pellets was provided to third and fourth instar larvae. Pupae were collected into 540 mL cylindrical plastic containers filled with water and placed in a cage (30 cm × 30 cm × 30 cm) for emergence. Emerging adults were sorted out by sex using morphological features. Battery operated handheld aspirators (Clarke^®^, St. Charles, IL, USA) were used by trained technicians to sort out the male mosquitoes from the cages within 24 h after emergence. Freshly emerged (<1 d old) male *Ae. aegypti* mosquitoes were placed in a small cup (266 mL) (200 mosquitoes per cup) to be treated at a 55 Gray (Gy) radiation dose using a Rad Source X-ray Machine (Rad Source RS 1800·Q, Rad Source Technologies, Buford, GA, USA) at a dose rate of 13.89 Gy/min for 7.8 min per round. This dosage was determined to be effective in sterilizing mosquitoes (>95% sterility) with minimal fitness cost for our mosquito colony [20]. After irradiation, male mosquitoes were allowed to rest for 30 min before being released back to their cages. Approximately 1000–1600 sterile males were kept per cage and provided 10% sucrose solution ad libitum until field released. Room temperature was maintained at 27 ± 2 °C, relative humidity of 55–65%, with a 12:12 h light:dark photo period. Sterile males were released into the field within 48 h after irradiation.

### 2.3. Selecting Sites for SIT Release

Historically high-count *Ae. aegypti* sites were selected for the SIT applications. The WVMVCD conducts weekly mosquito surveillance from February to November throughout its district. All sites were residential houses with typical housing structures. No permanent water body is adjacent to these sites. The Biogents (BG-2) Sentinel traps (Clarke, St. Charles, IL, USA) were complemented with synthetic lures that mimic human scents (Clarke, St. Charles, IL, USA), and a bucket filled with dry ice (1 kg) was utilized to monitor invasive *Aedes* mosquito abundance. Mosquitoes collected by these traps were sorted out into species and recorded.

Our routine IVM strategy for *Aedes* management includes source reduction, biological control using mosquitofish, and community outreach. When BG-2 traps indicate over 20 *Ae. aegypti*/trap-night, sites receive an In2Care^®^ Mosquito Station (In2Care.org, Wageningen, The Netherlands), which is kept at the site throughout the season. Here, a treatment site was defined as a residential property with an average property size of about 200 sq m (https://www.census.gov/programs-surveys/decennial-census/decade/2020/2020-census-results.html, accessed on 12 December 2024). Mosquito stations were serviced every four weeks. Details of the deployment and maintenance of In2Care^®^ Mosquito Stations have been previously reported [5].

A total of 25 *Ae. aegypti* hotspot sites (with >50 *Ae. aegypti* per trap-night during the peak mosquito season in 2023) were selected for SIT application. These sites were grouped into two cohorts—sites that received SIT treatment only (*n* = 9) and sites that received both SIT treatment and In2Care^®^ Mosquito Station (*n* = 16). In the latter group, sites received the In2Care^®^ Mosquito Station when the mosquito counts hit our set threshold (20 *Ae. aegypti*/trap-night), in accordance with our routine vector management protocol. Sites that received SIT treatment only did not have an In2Care^®^ Mosquito Station on the property or within a 0.5 km radius throughout the year to avoid its effect.

### 2.4. SIT Applications

Irradiated male *Ae. aegypti* were released within 48 h after irradiation treatment. Releases were made bi-weekly between April and November 2024, throughout the mosquito season. Between 1000 and 1600 sterile mosquitoes were kept in BugDorm cages (30 cm × 30 cm × 30 cm) (Insectabio Inc., Riverside, CA, USA), and releases were made in the morning between 10:00 and 11:30 h by opening the roof of the cage once the weather was warm enough for the mosquitoes to fly out. First, the technicians would canvass the area to make sure that the environment was optimal (e.g., not windy) for release. At each release, the number of dead mosquitoes in the cage was recorded. The number of mosquitoes released at each site was determined by the number of *Ae. aegypti* mosquitoes collected by the BG-2 traps in the week prior. At least 100-times the number of female *Ae. aegypti* mosquitoes (determined by BG-2 traps) were released at each site during the treatment weeks. Overall, between 1000 and 3000 sterile male *Ae. aegypti* were released at each site (1000–3000 sterile mosquitoes per 0.02 ha, which was approximately 50,000 per hectare) throughout the treatment weeks.

### 2.5. Quality Control

During the mosquito season, we extracted a subset of samples (*n* = 50) of irradiated male mosquitoes from the release cages and placed them in a separate cage with freshly emerged unirradiated female mosquitoes in a 1:1 ratio to evaluate the level of sterility of the eggs from these mosquitoes. This was undertaken to monitor the sterility of the irradiated male mosquitoes released. Sugar water (10%) was provided ad libitum. After one week, female mosquitoes were blood fed with bovine blood using glass feeders [19]. Mosquitoes were blood fed for two days. Five days after blood feeding, egg collection cups lined with filter paper and half filled with water and yeast infusion were placed in the cages and left for 5 days. Then, filter papers holding the eggs were removed and left to dry at normal room temperature. Once dried and counted, eggs were hatched using the procedure described above. Hatched larvae were counted, removed, and destroyed.

### 2.6. Follow-Up BG-2 Mosquito Trapping

Weekly mosquito surveillance was carried out at all SIT sites using BG-2 traps between April and November 2024. During the week of sterile insect release, trapping was conducted before the release day to avoid the chances of catching released mosquitoes. Traps were set on the front yard of houses under a tree or shrub in the afternoon and collected the following morning. Verbal consent was received from the homeowners before trapping at each residential site. Trapped mosquitoes were sorted to species under microscope by trained technicians using a morphological key [21]. The proportion of *Ae. aegypti* mosquitoes in traps ranged from 15 to 45% of the total collections. *Culex quinquefasciatus*, Cx. *tarsalis*, *Culiseta incidens*, and *Culiseta inornata* were also present in the traps.

### 2.7. Aedes-Related Service Requests

As an indirect way of measuring the impact of our vector control, we utilized the data of the service requests the WVMVCD received from residents within the district. A service request is defined as when residents call or email the district seeking mosquito management services when bothered by mosquitoes. As a government agency, it provides vector control services by sending out trained and licensed technicians to inspect the residential property and follow the IVM strategies as necessary to control mosquito breeding. Here, we collected the service request data for 2023 and 2024 to represent the pre-intervention and intervention periods, respectively. Service requests were considered *Aedes*-related if the resident complained about being bitten by mosquitoes during the day. If our optimized IVM strategies that incorporated SIT were working, we assumed that we would receive less service request calls.

### 2.8. Statistical Analysis

All recorded data were entered into Microsoft Excel and analyzed using SPSS version 20 (SPSS, Inc., Chicago, IL, USA). The mean number of female *Ae. aegypti* per trap-night was calculated for each cohort to compare the mosquito density pre-intervention and during intervention. Male counts were not included because there was no way to differentiate the released sterile insects from the wild ones. Therefore, efficacy of the sterile insect release application was measured by the number of female *Ae. aegypti* in the BG-2 traps. Data from 2023 were considered as “pre-intervention”, since no sterile insect releases were made in 2023. Since sterile releases were made throughout the 2024 mosquito season at these sites, the data from 2024 were considered as the “intervention” dataset. The monthly mean number of service requests was compared between the pre-intervention (2023) and intervention (2024) periods. These data were utilized as an indirect way to measure *Aedes*-related mosquito problems between the two study periods.

After testing the normality of the data using the Shapiro–Wilk test, repeated measures ANOVA was applied to determine differences in the mosquito density between the pre-intervention and intervention periods. All statistical analyses were performed at the 5% significance level.

## 3. Results

### 3.1. Sterile Ae. aegypti Releases

**Figure 2:**
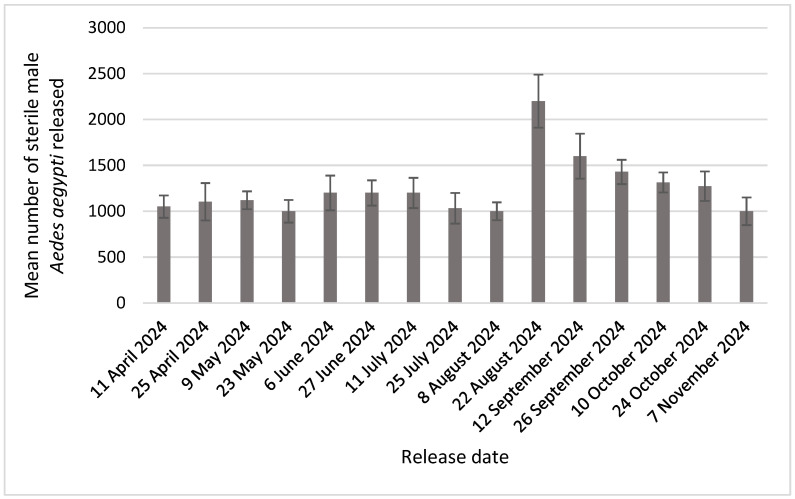
Mean number of sterile male *Aedes aegypti* mosquitoes released at each treatment site, April to November 2024.

A total of 106,608 sterile *Ae. aegypti* were released between April and November 2024 at 25 sites in the WVMVCD (Figure 2). At each site, on average, 1215 sterile mosquitoes (ranging from 1050 to 2200) were released bi-weekly. More sterile mosquitoes were released during the peak of the mosquito season (*p* < 0.05).

### 3.2. Effect of SIT on Mosquito Abundance

The mean number of female *Ae. aegypti* per trap-night was compared between the pre-intervention and intervention periods for both cohorts (Table 1). In the SIT treatment only cohort, the mean number of female *Ae. aegypti* per trap-night during the intervention period (mean = 10.8 female *Ae. aegypti* per trap-night; 95%CI = 8.5–13.1; ANOVA, *p* < 0.005) was 44% lower than in the pre-intervention period (mean = 19.2; 95%CI = 14.3–24.1). The cohort that received both In2Care^®^ Mosquito Stations and SIT treatment showed a 65% reduction in the number of female *Ae. aegypti* per trap-night in the intervention period (mean = 8.2; 95%CI = 3.6–12.8; ANOVA, *p* < 0.005) compared with the pre-intervention period (mean = 23.5; 95%CI = 19.1–27.9). During the intervention period, sites with the SIT treatment and In2Care Mosquito Stations had a 25% lower *Aedes* mosquito population than sites with the SIT treatment only.

### 3.3. Weekly Mosquito Abundance Trend

**Figure 3:**
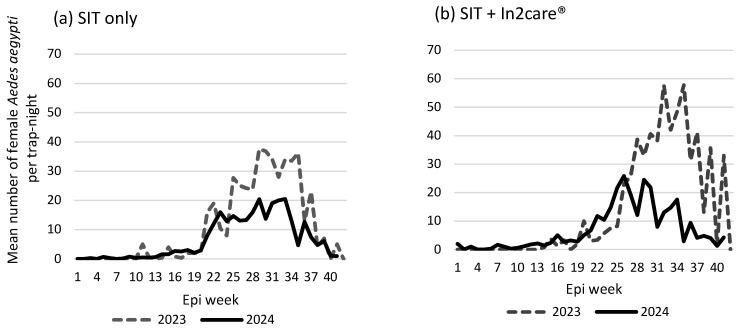
Weekly trend of female *Aedes aegypti* (mean number of female mosquitoes per trap-night) at the two cohorts—(**a**) sites that were treated with SIT only, and (**b**) sites that had In2Care^®^ Mosquito Stations and received SIT treatment.

The mean weekly number of female *Ae. aegypti* mosquitoes during the pre-intervention and intervention periods in the two cohorts is shown in Figure 3. The weekly female *Ae. aegypti* mosquito counts in both cohorts (SIT only and SIT sites with In2Care^®^ Mosquito Stations) were consistently lower in the intervention period compared with the pre-intervention period throughout most of the year. The highest reductions in mosquito counts during the intervention period were observed during the peak mosquito season (July and August).

### 3.4. Quality Control Data

**Figure 4:**
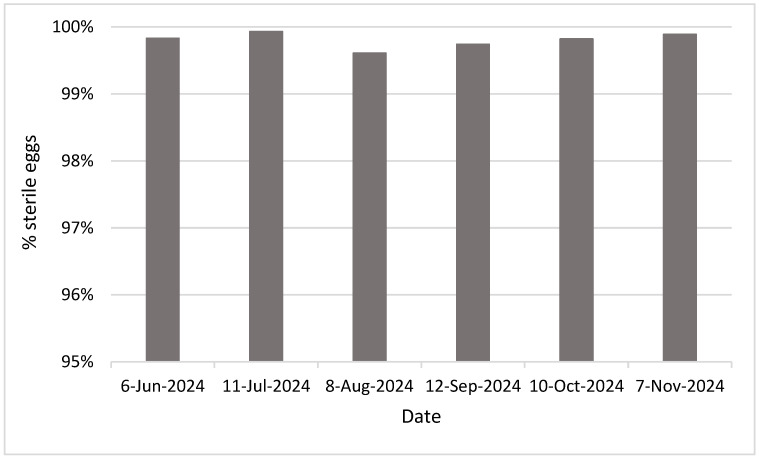
Quality control data—percentage of unhatched or sterile eggs from female *Aedes aegypti* mosquitoes that mated with the irradiated male mosquitoes.

A subset of sterile male *Ae. aegypti* mosquitoes from six releases were checked for sterility by letting them mate with unirradiated female mosquitoes in the laboratory. Over 99.6% of eggs (99.6 ± 0.13) collected from these female mosquitoes did not hatch, indicating a high level of sterility of the irradiated males utilized for our SIT application (Figure 4).

### 3.5. Aedes-Related Service Requests

**Figure 5:**
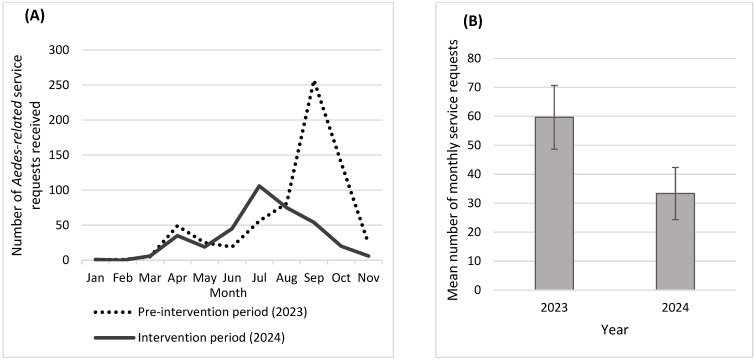
Monthly trend of *Aedes*-related service requests (**A**) and the mean number of monthly *Aedes*-related service requests (**B**) received during 2023 (pre-intervention) and 2024 (intervention period) in the West Valley Mosquito and Vector Control District.

The total number of *Aedes*-related service requests received from the district’s residents during the intervention year (2024) (*n* = 367) was 45% lower than the number of service requests received during the pre-intervention year (2023) (*n* = 656) (Figure 5). Especially during the peak mosquito season, the number of service requests dropped between 79 and 86% in September and October. While a slight increase in service requests was observed in July during the intervention year, the numbers remarkably declined thereafter throughout the mosquito season. Overall, the mean number of monthly *Aedes*-related service requests dropped significantly in the intervention period (mean = 33.4 service requests per month; 95%CI = 26.2–40.4; ANOVA, *p* < 0.05) compared with the pre-intervention period (mean = 59.6; 95%CI = 48.1–71.2).

## 4. Discussion

This study documented a significant reduction in the *Ae. aegypti* population in areas that received targeted bi-weekly SIT treatment. While SIT application alone resulted in a 44% reduction in the *Ae. aegypti* population at these hotspot sites, combining SIT with optimized IVM strategies (mainly In2Care^®^ Mosquito Stations) offered a 65% reduction in *Ae. aegypti* populations in treatment sites with historical *Aedes* endemicity. In addition, the number of service requests—residents seeking vector control services due to mosquito bites—dropped by 45% during the intervention period compared with the pre-intervention period. This highlighted the potential efficacy of a targeted SIT approach for invasive *Aedes* control in California. Unlike area-wide treatment, targeted SIT release is affordable and manageable, especially for small-to medium-sized vector control agencies, as it requires fewer resources. This is the first time that a significant invasive mosquito reduction was documented by utilizing a targeted SIT approach.

In Thailand, using a similar targeted approach—by releasing approximately 100–200 irradiated *Wolbachia*-infected *Ae. aegypti* males for six months in selected hotspot houses—a significant reduction (97.30%) in the mean number of female *Ae. aegypti* per household was achieved in the treatment areas when compared with the control ones [22]. In Greece, by releasing sterile male *Ae. aegypti* mosquitoes at a rate of 2547 ± 159 per hectare (or 10,000 sq·m) per week as part of an area-wide integrated vector management strategy, a gradual reduction in egg density, reaching 78% from mid-June to early September, was achieved [23]. In our study, we released an average of 1215 sterile mosquitoes (ranging from 1050 to 2200) per house (average property area = 200 sq·m) biweekly. Targeted SIT treatment is therefore impactful and convenient in areas with distinct invasive *Aedes* hotspots.

Tailoring the SIT program to meet the local circumstances is therefore vital for achieving the desired outcomes. A recent study reviewed 27 mark–release–recapture experiments and reported that the median distance travelled by *Ae. aegypti* is only 106 m [17]. This makes the targeted SIT application approach preferable, as released mosquitoes will remain in the treatment area. However, the effect of a resurgence in mosquitoes from adjacent mosquito prevalent sites could be inevitable [14,24]. In addition, there are studies that have documented a high dispersion rate of *Ae. aegypti* mosquitoes (512 m), which highlights the need to determine the flight ranges of local mosquitoes [25]. Therefore, continuous monitoring of the *Aedes* mosquito population by strengthening and expanding mosquito surveillance throughout the service area is crucial.

Between 2019 and 2024, the WVMVCD successfully doubled the annual number of mosquito traps set, increasing from 771 traps in 2016 to 1429 traps in 2024 (Supplementary Materials, Table S1). This significant expansion in surveillance efforts allowed us to monitor mosquito populations with greater precision and frequency. By intensifying our monitoring capabilities, we were able to identify mosquito hotspots in a timely manner, providing critical insights into where intervention efforts should be prioritized. Early detection of these hotspots, well before the mosquito populations peaked, enabled us to implement IVM strategies more comprehensively and effectively. This proactive approach ensured that resources were allocated efficiently, reducing the risk of outbreaks and contributing to better control of mosquito-borne diseases in the targeted regions.

Integrating In2Care^®^ Mosquito Stations and SIT into the IVM toolbox significantly enhances the control of *Aedes* mosquitoes by providing complementary, environmentally friendly tools. In2Care^®^ Mosquito Stations target both mosquito populations and their cryptic breeding habitats with minimal human intervention. The SIT, on the other hand, introduces sterile male mosquitoes into the population, which mate with wild females but produce no offspring, leading to a gradual population decline. Combining these methods within an IVM framework optimizes mosquito control by addressing multiple stages of the mosquito life cycle, enhancing the sustainability and effectiveness of control programs, reducing reliance on chemical insecticides, and mitigating the development of resistance. While the placement of In2Care^®^ Mosquito Stations is dependent on each homeowner’s voluntary approval in cities, which profoundly affects its widespread utilization, the SIT creates an opportunity to deal with mosquitoes by using the mosquitoes themselves [8]. Together, these tools offer a strategic advantage in combating *Aedes* populations and associated diseases such as dengue, chikungunya, and Zika virus [26,27].

The sterility of irradiated male *Ae. aegypti* reported in this study (>99.6%; Figure 4) was comparable to previous reports [20,28,29]. Such a high level of sterility highlights advancements in irradiation protocols and techniques, indicating the potential of sterile males in suppressing wild mosquito populations when released. High sterility rates combined with the high-irradiated mosquitoes’ survivorship (63% daily survivorship) were reported recently [20], enhancing the efficacy of the SIT applications. However, this needs to be tested in the field, since the data indicated here were only from laboratory colonies.

This study had some limitations. First, the data generated from this study were operational data. Since the WVMVCD is a government agency, we could not have control groups, as we are not allowed to deny interventions for any resident within our jurisdiction, which is why we did not have a control group without In2Care^®^ Mosquito Stations and SIT treatment. Other factors such as distance between treatment sites, the age of the released irradiated mosquitoes, and flight range of male mosquitoes should be determined in future studies.

In conclusion, as the SIT gains traction as a key tool in IVM strategies for *Ae. aegypti* control, targeted applications would benefit the effective utilization of limited resources. Here, we demonstrated the great potential of the SIT when combined with existing IVM strategies such as In2Care^®^ Mosquito Stations, offering a holistic approach to reducing the public health risks associated with *Aedes*-borne diseases. Extensive surveillance across the district allowed for the timely identification of *Aedes* hotspots, enabling targeted interventions at the beginning of the mosquito season before populations soared significantly. This proactive approach could help to effectively flatten the seasonal mosquito density curve, hence suppressing the mosquito population and associated risks. Importantly, the number of residents seeking vector control services declined during the intervention period compared with the pre-intervention period, reflecting the program’s success. Ensuring a high sterility rate of irradiated male mosquitoes through rigorous quality control measures proved essential in maintaining the efficacy of the SIT. Looking ahead, expanding efforts to identify and target *Aedes* hotspots intensively before the onset of the mosquito season will further suppress *Aedes* populations and minimize the risk of *Aedes*-borne diseases, enhancing public health outcomes across the district.

## Figures and Tables

**Table 1:** Mean number of female *Ae. aegypti* mosquitoes during the pre-intervention and intervention periods at two cohorts (sites that received SIT only, and those that had In2Care^®^ Mosquito Stations and received SIT treatment.

Cohort	No. of Sites	Pre-Intervention		Intervention		*p*-Value *	% Reduction
		No. Trap-Nights	Mean No. of Female *Ae. aegypti* per Trap Night (95%CI)	No. Trap-Nights	Mean no. of Female *Ae. aegypti* per Trap Night (95%CI)		
SIT only	9	155	19.2 (14.3–24.1)	229	10.8 (8.5–13.1)	<0.001	44%
SIT with In2Care^®^ Mosquito Stations	16	193	23.5 (19.1–27.9)	288	8.2 (3.6–12.8)	<0.001	65%

* The difference in the mean number of female *Ae. aegypti* per trap-night was compared by one-way ANOVA.

## Data Availability

The original contributions presented in this study are included in the article; further inquiries can be directed to the corresponding author.

## Supplementary Materials

The following supporting information can be downloaded at: https://www.mdpi.com/article/10.3390/insects16010081/s1, Table S1: Annual mean number of Ae aegypti mosquitoes per trap-night and total BG trap-nights set across the West Valley Mosquito and Vector Control District, California.
