# Supplementary material for: Reduction in Aedes aegypti Population After a Year-Long Application of Targeted Sterile Insect Releases in the West Valley Region of Southern California
Source: Insects. 2025 Jan 15;16(1):81. doi: 10.3390/insects16010081 (PMC11765725; doi:10.3390/insects16010081)

Supplementary Table: Annual mean number of Ae aegypti mosquitoes per trap-night and total BG trap-nights set across the West Valley Mosquito and Vector Control District, California.

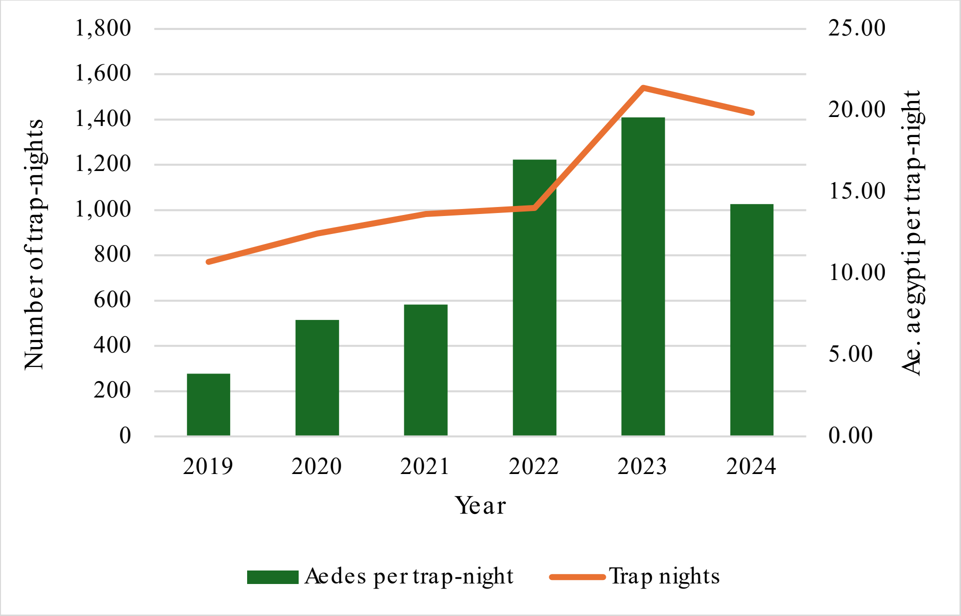

Supplement: Supplementary file 1 [file insects-16-00081-s001.zip › insects-3408407-supplementary.pdf]
